# Estimating the Disease Burden of Pandemic (H1N1) 2009 Virus Infection in Hunter New England, Northern New South Wales, Australia, 2009

**DOI:** 10.1371/journal.pone.0009880

**Published:** 2010-03-25

**Authors:** Fatimah S. Dawood, Kirsty G. Hope, David N. Durrheim, Rodney Givney, Alicia M. Fry, Craig B. Dalton

**Affiliations:** 1 Influenza Division, Centers for Disease Control and Prevention (CDC), Atlanta, Georgia, United States of America; 2 Hunter New England Population Health and Newcastle Institute of Public Health, University of Newcastle, Wallsend, New South Wales, Australia; 3 Hunter New England Population Health and Hunter Medical Research Institute, Wallsend, New South Wales, Australia; 4 Hunter Area Pathology Service, John Hunter Hospital, Newcastle, New South Wales, Australia; University of Oxford, United Kingdom

## Abstract

**Introduction:**

On May 26, 2009, the first confirmed case of Pandemic (H1N1) 2009 virus (pH1N1) infection in Hunter New England (HNE), New South Wales (NSW), Australia (population 866,000) was identified. We used local surveillance data to estimate pH1N1-associated disease burden during the first wave of pH1N1 circulation in HNE.

**Methods:**

Surveillance was established during June 1-August 30, 2009, for: 1) laboratory detection of pH1N1 at HNE and NSW laboratories, 2) pH1N1 community influenza-like illness (ILI) using an internet survey of HNE residents, and 3) pH1N1-associated hospitalizations and deaths using respiratory illness International Classification of Diseases 10 codes at 35 HNE hospitals and mandatory reporting of confirmed pH1N1-associated hospitalizations and deaths to the public health service. The proportion of pH1N1 positive specimens was applied to estimates of ILI, hospitalizations, and deaths to estimate disease burden.

**Results:**

Of 34,177 specimens tested at NSW laboratories, 4,094 (12%) were pH1N1 positive. Of 1,881 specimens from patients evaluated in emergency departments and/or hospitalized, 524 (26%) were pH1N1 positive. The estimated number of persons with pH1N1-associated ILI in the HNE region was 53,383 (range 37,828–70,597) suggesting a 6.2% attack rate (range 4.4–8.2%). An estimated 509 pH1N1-associated hospitalizations (range 388–630) occurred (reported: 184), and up to 10 pH1N1-associated deaths (range 8–13) occurred (reported: 5). The estimated case hospitalization ratio was 1% and case fatality ratio was 0.02%.

**Discussion:**

The first wave of pH1N1 activity in HNE resulted in symptomatic infection in a small proportion of the population, and the number of HNE pH1N1-associated hospitalizations and deaths is likely higher than officially reported.

## Introduction

On May 26, 2009, the first confirmed case of infection with Pandemic (H1N1) 2009 virus (pH1N1) in a resident of Hunter New England (HNE) Area Health Service, Australia was identified. HNE is a region located in the northeast of the Australian state of New South Wales (NSW) which covers a geographic area of 130,000 square kilometers and has a population of approximately 866,000 people whose public health needs are served by Hunter New England Population Health (HNEPH) [1]. Upon identification of the introduction of pH1N1 to Australia, HNEPH rapidly established multiple surveillance systems to track and determine the impact of circulation of the pandemic virus in the community. Use of available influenza syndromic surveillance and virologic surveillance data has been proposed as one method to quantify disease burden in a pandemic when cases of infection rise rapidly and disease burden can no longer be estimated using confirmed case counts [2]. We used data from complementary surveillance systems in HNE to estimate the disease burden resulting from pH1N1 circulation and to describe the first wave of pH1N1 activity, including laboratory detection of pH1N1 virus, community influenza-like illness, hospitalizations, and deaths, over a three month period from June 1-August 30, 2009.

## Materials and Methods

### Laboratory Confirmed pH1N1 Virus Infection

**Table 1:** Data Sources Used For Pandemic (H1N1) 2009 Disease Burden Estimates.

	Data Sources
**Community ILI**	1) FluTracking syndromic surveillance for influenza-like illness
	2) New South Wales virologic data
**Hospitalizations**	1) Reported hospitalizations
	2) ICD-10 acute respiratory illness hospitalization surveillance
	3) HAPS virologic data from ED and hospitalized patients
**Deaths**	1) Reported deaths
	2) ICD-10 acute respiratory illness deaths surveillance
	3) HAPS virologic data from ED and hospitalized patients

HAPS: Hunter Area Pathology Service; ED: Emergency Department.

#### New South Wales Virologic Data

The two reference laboratories in NSW are South Eastern Area Laboratory Services (SEALS) and Institute of Clinical Pathology and Medical Research (ICPMR) based in Sydney. NSW Department of Health conducted influenza virologic surveillance at SEALS, ICPMR, and five NSW government operated laboratories. NSW surveillance laboratories received specimens taken from persons seeking medical care in NSW, and results of influenza diagnostic testing (excluding rapid antigen and serologic testing) were published on the internet each week [3]. Using the results of influenza diagnostic tests at NSW surveillance laboratories during May 30–August 28, we calculated the weekly proportion of respiratory specimens tested for influenza viruses that were positive for pH1N1 virus, with 95% confidence intervals for a standard distribution based on the number of specimens tested. Specimens included those submitted by general practitioners evaluating persons presenting for outpatient care or from clinicians treating Emergency Department (ED) or hospitalized patients.

#### Hunter Area Pathology Service Virologic Data

Hunter Area Pathology Service (HAPS) is one of the five commercial laboratories participating in NSW surveillance and provides influenza diagnostic testing for 31 of the 35 hospitals in the HNE area with EDs where patients with respiratory illness are evaluated. HAPS also provides diagnostic testing for a number of hospitals in northern and western NSW. During June 1–14, 2009, all respiratory specimens that were positive for influenza A viruses by real time reverse transcriptase polymerase chain reaction (rRT-PCR) at HAPS were sent to the two state reference laboratories in Sydney for further testing for pH1N1. On June 15, 2009, HAPS began rRT-PCR testing for pH1N1, and subsequently, the majority of local respiratory specimens were tested by HAPS for influenza A and B, and specimens that were found to be positive for influenza A were tested for pH1N1. Using specimens from patients seen in EDs and/or hospitalized at HNE hospitals, we calculated the weekly proportion of specimens that were positive for pH1N1 virus infection with 95% confidence intervals assuming a standard distribution for June 15-August 30, 2009.

### Community Illness Surveillance

FluTracking is an on-line community health syndromic surveillance system established in 2006 which monitors rates of influenza-like illness (ILI) (defined as the presence of self-reported fever and cough) among residents of all six states and two territories in Australia [4], [5]. Many FluTracking participants in Hunter New England are employees or family members of employees of Hunter New England Area Health Service. During each week of the influenza season, from May through October, FluTracking participants receive a weekly email with a hyperlink to their FluTracking web-based accounts encouraging them to complete a brief, 10–15 second survey collecting information about the presence of ILI during the previous week. FluTracking participants may enroll and complete surveys for other members of their households, and enrollment continues throughout the influenza season. At the start of the 2009 influenza season, there were approximately 1,800 individual FluTracking participants in HNE accounting for approximately 0.2% of the HNE population.

To calculate the weekly point estimate of FluTracking participants with pH1N1-associated influenza-like illness (ILI), we multiplied the weekly point estimate of the proportion of specimens positive for pH1N1 virus from NSW virologic data by the weekly number of FluTracking participants reporting ILI:
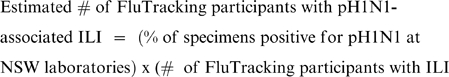



To calculate low and high estimates of FluTracking participants with pH1N1-associated ILI, we performed the same calculation with the bounds of the 95% confidence intervals of the proportion of NSW laboratory specimens positive for pH1N1 virus. To determine the weekly estimate of HNE residents with pH1N1-associated ILI, we multiplied the weekly estimate of FluTracking participants with pH1N1-associated ILI by the population of HNE (866,565 persons per 2008 Australian census data) divided by the number of HNE FluTracking participants who completed a survey for the week:
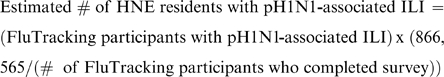



### Hospitalization Surveillance and Burden Estimates

#### Reported Hospitalizations

During the 2009 influenza season, hospitals were asked to report all persons hospitalized with confirmed pH1N1 infection to HNEPH. Reported cases were entered into a web-based database called NetEpi. Reported cases with a positive test date from June 1–August 30, 2009 were identified through review of data from the NetEpi database.

#### Estimated Hospitalizations

In addition, HNEPH established population-based surveillance for hospitalizations due to respiratory illness at all 35 HNE hospitals with emergency departments (EDs). Hospitalizations were identified based on one of eight ED International Classification of Diseases 10 (ICD-10) discharge codes (Table 2). The eight ICD-10 codes were chosen by retrospectively reviewing coding practices for respiratory illness at the 35 HNE hospitals during previous influenza seasons to select the most frequently used codes during previous seasons. Of patients admitted for any illness to the 35 surveillance hospitals during 2002–2007 (n = 160,204 to 179,132), 96% were residents of HNE each year. Therefore, we assumed that 96% of persons admitted for respiratory illness in HNE during the 2009 influenza season were residents of HNE.

**Table 2:** Emergency Department ICD-10 Codes Used For Surveillance for Hospitalizations and Deaths Due To Acute Respiratory Illness.

Code	Diagnosis	% of Hospitalized Cases (N = 2113 )	% of Death Cases (N = 50)
J11	Influenza, virus not identified	0%	0%
J11.1	Influenza with other respiratory manifestations, virus not identified	27%	2%
J11.8	Influenza with other manifestations, virus not identified	25%	0%
J12.9	Viral pneumonia, unspecified	1%	0%
J15.9	Bacterial pneumonia, unspecified	1%	0%
J18.9	Pneumonia, unspecified	32%	72%
J22	Unspecified acute lower respiratory infection	12%	6%
J96.9	Respiratory failure, unspecified	2%	20%

To determine a weekly point estimate of pH1N1-associated hospitalizations among HNE residents, we multiplied the proportion of specimens positive for pH1N1 virus from HAPS virologic data by the weekly number of hospitalizations identified through the ICD-10 surveillance system:
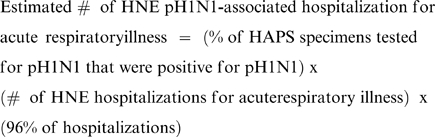



To determine low and high estimates of the weekly number of pH1N1-associated hospitalizations among HNE residents, we performed the same calculation with the bounds of the 95% confidence intervals of the proportion of HAPS specimens positive for pH1N1. During June 1-June 14, HAPS data on the proportion of specimens positive for pH1N1 were not available because HAPS was not performing the pH1N1 rRT-PCR assay. Therefore, we substituted the proportion positive from NSW laboratory data for the period June 1-June 14 for the above calculation and the calculation of the estimated number of pH1N1-associated deaths described below.

### Mortality Surveillance and Burden Estimate

#### Reported Deaths

During the 2009 influenza season, hospitals and coroners were asked to report all persons who died with confirmed pH1N1 infection to HNEPH, and reported cases were entered into NetEpi. Reported persons with a death date from June 1-August 30, 2009 were identified through review of data from the NetEpi database.

#### Estimated Deaths

In addition, HNEPH established population-based surveillance for deaths due to respiratory illness at the same 35 HNE hospitals included in the ICD-10 hospitalization surveillance system. Deaths were identified by reviewing hospital death lists and identifying patients with the same eight ICD-10 ED discharge codes for acute respiratory illness used for hospitalization surveillance (Table 2). As with hospitalizations, we assumed that 96% of deaths identified through hospitalization surveillance were residents of HNE. To determine a point estimate of the weekly number of pH1N1-associated deaths among HNE residents, we multiplied the proportion of specimens positive for pH1N1 from HAPS data by the weekly number of HNE respiratory illness deaths identified through the surveillance system:
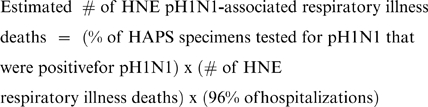



Deaths with confirmed pH1N1 infection which occurred in residents of aged and long- term care facilities are not identified through this surveillance system. Therefore, deaths with confirmed pH1N1 infection reported to HNEPH from these facilities were added to the weekly total number of pH1N1-associated deaths.

### Human Subjects Review

Surveillance data and laboratory data used in this analysis were collected as part of public health response, and thus, data collection was not subject to institutional review board approval for human research protections. Patient identifiers were not included in data collection, and all data were analyzed in aggregate.

## Results

### Virologic Data

During May 30–August 28, 2009, 34,177 specimens were tested for influenza viruses by SEALS, ICPMR, and five NSW pathology services, and of those, 7,485 (22%) were positive for influenza A virus, and 4,094 specimens (12% of all specimens tested for influenza viruses, 55% of specimens testing positive for influenza A viruses) were positive for pH1N1. The proportion of specimens positive for pH1N1 increased from 0.8% (CI 0–0.8%) (during May 30–June 5) to 22% (CI 20–24%) (during June 27–July 3) (Table 3) and subsequently declined.

**Table 3:** Incidence of Specimens Positive for Pandemic (H1N1) 2009 At New South Wales (NSW)[1] and Hunter Area Pathology Service (HAPS) Laboratories, June 1-August 30, 2009.

		NSW Specimens		HAPS Specimens	
		(N = 34,177)		(N = 1,881)	
Week*		n	% Positive for pH1N1	n	% Positive for pH1N1
1	6/1–6/7/2009	2157	1% (0–1%)	–	–
2	6/8–6/14/2009	2886	3% (3–4%)	–	–
3	6/15–6/21/2009	3615	6% (5–7%)	30	3% (0–9%)
4	6/22–6/28/2009	3478	9% (8–10%)	84	14% (7–21%)
5	6/29–7/5/2009	2824	22% (20–24%)	130	23% (16–30%)
6	7/6–7/12/2009	3499	19% (18–20%)	231	26% (20–32%)
7	7/13–7/19/2009	3356	18% (17–19%)	341	27% (22–32%)
8	7/20–7/26/2009	3755	17% (16–18%)	302	36% (31–41%)
9	7/27–8/2/2009	2637	16% (15–17%)	257	34% (28–40%)
10	8/3–8/9/2009	1918	9% (8–10%)	193	24% (18–30%)
11	8/10–8/16/2009	1499	9% (8–10%)	162	23% (17–29%)
12	8/17–8/23/2009	1424	7% (8–9%)	151	23% (16–30%)
13	8/24–8/30/2009	1129	5% (4–6%)	136	13% (7–19%)

*Dates reflect start and end dates for weekly HAPS laboratory data. Weekly NSW laboratory data were available for weeks with a start and end date 2 days prior to the dates for HAPS laboratory data (for example, week 1 5/30–6/5/2009).

1. Weekly Influenza Epidemiology Report, NSW. 2009. (Accessed September 8, 2009, 2009, http://www.emergency.health.nsw.gov.au/swineflu/resources/pdf/case_statistics_020909.pdf.).

During June 15–August 30, 2009, 1,881 specimens from patients evaluated in HNE emergency departments and/or hospitalized in HNE hospitals were submitted to HAPS laboratories for testing for influenza viruses by rRT-PCR; of those specimens, 725 (39%) were positive for influenza A viruses, and 524 (26% of all specimens tested for influenza viruses, 72% of specimens testing positive for influenza A viruses) were positive for pH1N1. The proportion of HAPS specimens positive for pH1N1 increased from 3% to 36% during weeks 3–8 of the outbreak (June 15–July 26) and peaked at 36% and 34% during weeks 8 and 9 of the outbreak (July 20–August 2) (Table 3).

### Community Illness

**Figure 1:**
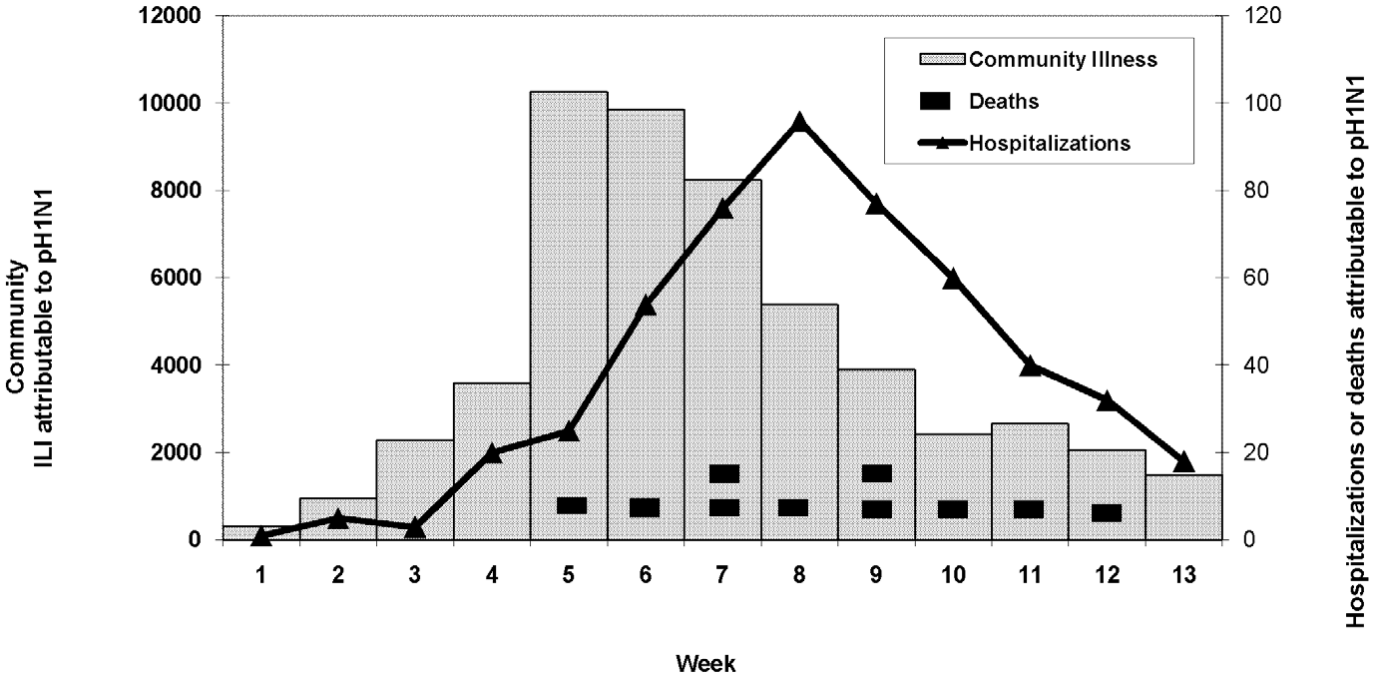
Community ILI, Hospitalizations, and Deaths Associated with Pandemic (H1N1) 2009 Virus Infection, Hunter New England, New South Wales, June 1-August 30, 2009.

During June 1–August 30, 2009, the estimated number of persons with pH1N1-associated ILI in HNE was 53,383 (range 37,828–70,597) suggesting a pH1N1 attack rate of 6.2% (range 4.4–8.2%) (Table 4). The number of persons with pH1N1-associated ILI peaked during week 5 of the outbreak (June 29–July 5), remained elevated during weeks 6 and 7 of the outbreak (July 6–19), and then declined during subsequent weeks (Figure 1).

**Table 4:** Reported and Estimated Pandemic (H1N1) 2009 Disease Burden, June 1–August 30, 2009, Hunter New England, New South Wales.

	Reported Number	Calculated Number	Estimated Rate Per 100,000*	Case Ratio**
**Deaths**	5	10 (8–13)	0.6 to 1.5	0.009% to 0.02%
**Hospitalizations**	184	509 (388–630)	20 to 73	0.3% to 1%
**Community Illness**	---	53,383 (37,828–70,597)	4,365 to 8,147	---

*Rate ranges calculated using reported number (when available) and upper bound of calculated number.

**Case fatality and case hospitalization ratio ranges calculated using reported number and calculated point estimate of deaths and hospitalizations divided by calculated point estimate of community illness.

### Hospitalizations

**Figure 2:**
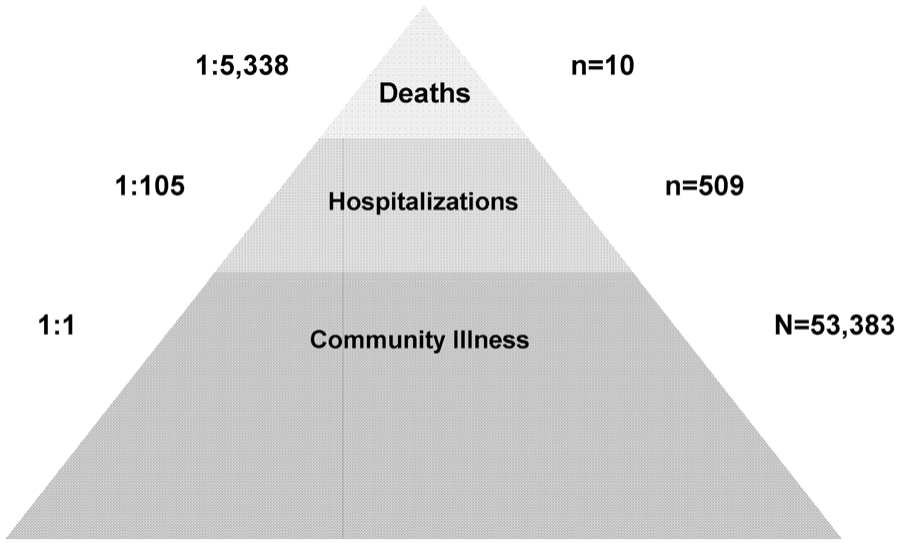
Estimated Pandemic (H1N1) 2009 Disease Burden, Hunter New England, New South Wales, June 1-August 30, 2009.

During June 1–August 30, 2009, 184 persons hospitalized with confirmed pH1N1 infection were reported to HNEPH, and 509 pH1N1-associated hospitalizations (range 388–630) were estimated using ICD-10 hospitalization surveillance data. The estimated number of pH1N1-associated hospitalizations increased rapidly during weeks 5–8 of the outbreak (June 29–July 26) and peaked during week 8 of the outbreak (July 20–26) (Figure 1). Based on reported hospitalizations and calculated estimates using hospitalizations identified through ICD-10 surveillance, the rate of pH1N1-associated hospitalization ranged from 21 to 73 hospitalizations per 100,000 persons. Based on point estimates of pH1N1-associated community illness and pH1N1-associated hospitalizations, approximately 1 in 105 persons with pH1N1 infection was hospitalized resulting in a case hospitalization ratio of 1% (Figure 2).

### Deaths

During June 1-August 30, 2009, five deaths reported to HNEPH had confirmed pH1N1 infection (none occurred in aged- or long-term care facilities, but one occurred in the community with pH1N1 infection confirmed at autopsy), and 10 deaths (range 8–13) associated with pH1N1 infection were estimated using ICD-10 mortality surveillance data. Using the reported number of deaths and the point estimate of deaths compared to the point estimate of community illness, the case fatality ratio (CFR) ranged from 0.009% for reported, confirmed pH1N1-associated deaths to 0.02% for estimated deaths.

## Discussion

The first wave of infection with pH1N1 among residents of Hunter New England, New South Wales resulted in an estimated 53,383 influenza-like illnesses, 509 hospitalizations, and up to 10 deaths among the approximately 866,000 residents of HNE during a 13 week period. Based on these estimates, approximately 1 in 16 HNE residents had symptomatic pH1N1 infection during the surveillance period, and 1 in 1700 HNE residents required hospitalization for pH1N1-associated illness.

Compared to the typical influenza season in HNE which occurs from June through October and peaks in August or September, the first wave of pH1N1 activity began and peaked earlier during a three week period in early July. A similar trend towards an earlier peak in the influenza season was also observed in the southern Australian state of Victoria [6]. Compared to the peak of pH1N1 community illness in HNE, the peak in pH1N1-associated hospitalizations lagged by 1–2 weeks which may be due in part to a delay between illness onset and presentation for medical care and varied transmission patterns influenced by social networks with initial transmission among healthy children and later transmission among more vulnerable populations. While the burden of community illness and pH1N1-associated hospitalizations in HNE increased rapidly resulting in clear peaks, pH1N1-associated deaths occurred more sporadically.

In this analysis, the pH1N1 community ILI attack rate ranged from 4–8%, similar to the estimated attack rate from New Zealand (8%)[7] which experienced the introduction of pH1N1 during the Southern Hemisphere influenza season. During May through August, 2009, outbreaks of pH1N1 infection occurred throughout the United States outside of the typical Northern Hemisphere influenza season, and several estimates of ILI attack rate were made. United States all-cause ILI attack rate estimates ranged from 5% for a four week period in 10 states[8] to 7% for a four week period in New York City [9]. In contrast to these estimates from the United States, our estimate was adjusted using virologic surveillance data suggesting that the ILI attack rate in HNE during the influenza season was higher than in parts of the United States where estimates were made when pH1N1 was circulating outside of the United States influenza season. Attack rates from our analysis, as well as those from most other analyses, underestimate the true pH1N1 attack rate, because asymptomatic infections are not included.

During the twentieth century, three influenza pandemics occurred starting in 1918, 1957, and 1968. Of the twentieth century pandemics, the 1918 pandemic was the most severe with estimated attack rates for the full pandemic period ranging from 20–60% and estimated case fatality ratios ranging from 2–3% [10], [11]. In New South Wales, the 1918 pandemic virus was estimated to result in 300 deaths per 100,000 persons [11]. In comparison, the 1957 and 1968 pandemics were milder with case fatality ratios estimated at less than 0.2% [10]. Our estimated community attack rate of 4–8% and estimate of approximately 1 death per 100,000 persons with a case fatality ratios of 0.009–0.02% suggest that compared to the 20^th^ century pandemics, the first wave of the current pandemic resulted in clinical infection in a smaller proportion of the population and in fewer severe outcomes. However, our estimates reflect only the first wave of symptomatic pH1N1 activity while estimates from prior pandemics frequently reflect the result of multiple waves of pandemic virus activity. Thus, it remains difficult to predict the overall disease burden that will result from pH1N1.

Our model for estimating pH1N1 disease burden in HNE has several strengths. First, because the Hunter New England community is a well- defined population with a limited number of hospitals where persons with acute respiratory illness are hospitalized, HNEPH was able to implement population-based surveillance for acute respiratory illness hospitalizations and deaths, eliminating the need to extrapolate estimates from a limited sample of the population. HNE hospitals also maintained a consistent catchment area allowing us to easily adjust for hospitalizations of non-HNE residents prior to calculating hospitalization and mortality rates. Second, as surveillance for community illness was internet-based and surveillance for hospitalizations and deaths was based on ICD-10 codes from electronic medical record systems, surveillance data was readily available allowing for a rapid estimate of disease burden and severity.

The limitations of our model reflect the constraints inherent in each of the surveillance systems used for estimating each measure of pH1N1 burden: detection of pH1N1, community illness, hospitalizations, and deaths. First, both sources of laboratory data were based on specimens collected and sent at the discretion of the evaluating physician, and thus were affected by clinician testing practices. In particular, the NSW laboratory data used to estimate the incidence of pH1N1 in the community may have been affected by the shift in the Australian pandemic phase from CONTAIN to PROTECT on June 17, 2009 [12], after which clinicians were advised to focus testing on hospitalized patients and on persons with characteristics conferring a higher risk for severe influenza. However, given the number of specimens submitted for testing at the two NSW reference laboratories and five pathology services compared to the reported number of hospitalizations in NSW [3], it is likely that the majority of specimens were taken from non-hospitalized persons. We also assumed that the incidence of pH1N1 infection was similar among persons with respiratory illness who sought outpatient medical care and among persons with respiratory illness who did not seek medical care. Second, since the majority of participants in the FluTracking surveillance system are employees of the HNE Area Health Service, FluTracking participants may differ from the general HNE population with respect to socioeconomic status and educational background which would affect our estimates of the burden of pH1N1-associated community ILI if these factors were associated with influenza transmission. Third, our estimates of pH1N1-associated hospitalizations and deaths are based on surveillance for hospitalizations and deaths using ICD-10 codes for respiratory illness which are less likely to capture persons with pH1N1 who have cardiovascular presentations or exacerbations of underlying illnesses. An analysis of ICD-10 codes assigned to reported, confirmed case-patients with pH1N1-associated hospitalizations admitted from HNE EDs found that 50% of reported cases were captured by one of the surveillance ICD-10 codes, while the remaining 50% of cases were assigned a broad range of codes [unpublished data], making it difficult for syndromic surveillance to capture these undetected cases while achieving adequate specificity. Surveillance ICD-10 codes also were selected by retrospective review of codes used during prior influenza seasons when coding practices may have been different than during the current pandemic. Lastly, it should be noted that virologic data from ED patients and hospitalized patients was used to calculate our estimates of pH1N1-associated deaths. It is unclear how this might bias our estimates if the proportion of persons with pH1N1 differed among persons who were hospitalized with respiratory illness and those who died with respiratory illness.

This analysis was also unable to explore age-specific differences in disease burden because the number of hospitalizations and deaths was relatively small resulting in small numbers in each age stratum. Seasonal influenza community attack rates and hospitalization and mortality rates have been shown to vary substantially by age [13], [14], [15]. In addition, the age distribution of confirmed pH1N1 cases from many countries suggests that people aged 65 years and older may be at lower risk for pH1N1 infection, and are underrepresented among pH1N1-associated hospitalizations and deaths when compared to seasonal influenza [16], [17], [18]. In this analysis, approximately 20% of hospitalizations and 84% of deaths identified through ICD-10 surveillance occurred in persons aged 65 years and older. If the incidence of pH1N1 infection is lower among persons aged older than 65 years, then we may have over-estimated deaths, and possibly hospitalizations, by applying non-age-specific virologic data to hospitalizations identified through ICD-10 surveillance. However, despite this potential limitation, our estimated case fatality ratio is relatively low compared to prior pandemics and consistent with estimates from other countries [7], [19].

We estimate that pH1N1 had an attack rate of 4–8% and a case fatality ratio of 0.009–0.02% during the first wave of pH1N1 activity in HNE, Australia. Our estimates are consistent with estimated attack rates from New Zealand which experienced the introduction of pH1N1 during the Southern Hemisphere influenza season but may be higher than estimates from the first wave of pH1N1 activity in the United States where pH1N1 introduction occurred outside of the influenza season. It remains to be seen whether a second wave of pH1N1 activity will occur in HNE during 2010 and whether the characteristics of the pandemic virus and its host population will change resulting in a different pattern of pH1N1 disease burden in HNE as the worldwide pandemic progresses.

## References

[1] About Hunter New England Health. Accessed September 22, 2009 at http://www.hnehealth.nsw.gov.au/about_us

[2] Lipsitch M, Hayden FG, Cowling BJ, Leung GM (2009). How to maintain surveillance for novel influenza A H1N1 when there are too many cases to count.. Lancet.

[3] Weekly Influenza Epidemiology Report, NSW. Accessed August 30, 2009 at http://www.health.nsw.gov.au/news/2009/20090827_01.html

[4] Flutracking.net. Accessed on September 22, 2009 at www.flutracking.net.

[5] Parrella A, Dalton C, Pearce R, Litt J (2009). ASPREN surveillance system for influenza-like illness: A comparison with FluTracking and the National Notifiable Diseases Surveillance System.. Australian Family Physician.

[6] The 2009 Victorian Influenza Vaccine Effectiveness Report. Accessed September 2, 2009, at http://www.vidrl.org.au/surveillance/flu%20reports/flurpt09/pdf_files/flu0918.pdf

[7] Baker MG, Wilson N, Huang QS, Paine S, Lopez L (2009). Pandemic influenza A(H1N1)v in New Zealand: the experience from April to August 2009.. Euro Surveill.

[8] Reed C, Angulo F, Swerdlow D, Meltzer M, Jernigan D (2009 (Epub ahead of print)) Estimates of the prevalence of pandemic (H1N1) 2009, United States, April-July 2009 Emerg Infect Dis.

[9] Prevalence of Flu-Like Illness in New York City: May 2009. (2009). http://www.nyc.gov/html/doh/downloads/pdf/cd/h1n1_citywide_survey.pdf.

[10] Pandemic influenza preparedness and response: a WHO guidance document..

[11] Mathews JD, Chesson JM, McCaw JM, McVernon J (2009). Understanding influenza transmission, immunity and pandemic threats.. Influenza Other Respi Viruses.

[12] Media release: new pandemic phase PROTECT. Accessed September 2, 2009 at http://www.healthemergency.gov.au/internet/healthemergency/publishing.nsf/Content/resources/File/090617%20protect%20media%20release%20FINAL.pdf

[13] Fox JP, Cooney MK, Hall CE, Foy HM (1982). Influenza virus infections in Seattle families, 1975-1979. II. Pattern of infection in invaded households and relation of age and prior antibody to occurrence of infection and related illness.. Am J Epidemiol.

[14] Mullooly JP, Bridges CB, Thompson WW, Chen J, Weintraub E (2007). Influenza- and RSV-associated hospitalizations among adults.. Vaccine.

[15] Neuzil K, Mellen B, Wright P, Mitchel E, Griffin M (2000). The Effect of Influenza on Hospitalizations, Outpatient Visits, and Courses of Antibiotics in Children.. The New England Journal of Medicine.

[16] Assessment of the 2009 influenza A (H1N1) pandemic on selected countries in the southern hemisphere: Argentina, Australia, Chile, New Zealand, Uruguay. Accessed September 5, 2009 at http://www.flu.gov/professional/global/southhemisphere.html.

[17] Australian Influenza Surveillance Report, No. 11, 2009. http://www.healthemergency.gov.au/internet/healthemergency/publishing.nsf/Content/18D06BAC4644C98DCA25763E00823442/File/ozflu-no11-2009.pdf.

[18] ECDC interim risk assessment: Pandemic H1N1 2009. http://ecdc.europa.eu/en/healthtopics/Documents/0908_Influenza_AH1N1_Risk_Assessment.pdf.

[19] Wilson N, Baker MG (2009). The emerging influenza pandemic: estimating the case fatality ratio.. Euro Surveill.

